# Clinical and ultrasound findings of pentalogy of Cantrell in a newborn: A case report

**DOI:** 10.3389/fped.2022.998495

**Published:** 2022-11-15

**Authors:** Dan Wang, Bin Zheng, Bo Zhai, Juan Mo, Kaihua Yang, Yaling Huo

**Affiliations:** ^1^ Department of Ultrasound, Children's Hospital Affiliated to Zhengzhou University, Henan Children's Hospital, Zhengzhou Children's Hospital, ZhengZhou China; ^2^ Department of Radiology, Children's Hospital Affiliated to Zhengzhou University, Henan Children's Hospital, Zhengzhou Children's Hospital, ZhengZhou China; ^3^ Department of Cardiothoracic Surgery, Children's Hospital Affiliated to Zhengzhou University, Henan Children's Hospital, Zhengzhou Children's Hospital, ZhengZhou China

**Keywords:** pentalogy of Cantrell, ultrasound, ventricle, diverticulum, case report

## Abstract

**Background:**

Pentalogy of Cantrell is a rare and deadly syndrome, manifesting as intracardiac anomalies and ventricular diverticulum. Echocardiographers have an insufficient understanding of pentalogy of Cantrell, which may lead to missed diagnoses, especially in cases lacking the most obvious signs.

**Case summary:**

One of twin male infants, at a gestational age of 37 weeks, was found with a cardiac murmur and a pulsatile mass in the midline supraumbilical abdomen for 2 days. Echocardiography on admission indicated congenital heart disease. A cardiac murmur was detected in the 3–4 intercostal space and extensively spread. The infant was diagnosed with pentalogy of Cantrell by ultrasound and computed tomography angiography (CTA) preoperatively. The patient underwent heart deformity surgery and was followed up for 16 months. The patient's cardiac structure and function returned to normal.

**Conclusion:**

Intracardiac anomaly and ventricular diverticulum are the primary manifestations of pentalogy of Cantrell. Pentalogy of Cantrell may be diagnosed by combining the ultrasound and CTA findings.

## Introduction

Pentalogy of Cantrell is a collection of congenital malformations involving the heart, pericardium, diaphragm, sternum, and ventral abdominal wall and was first reported by Cantrell et al. (1). The prevalence of the pathology is <1 per 100,000 live births (2, 3). This syndrome comprises five abnormalities: lower sternum anomaly; deficiency of the anterior diaphragm; a defect in the pericardium; midline supraumbilical abdominal wall defect; and intracardiac anomaly with ectopia cordis, which is divided into complete and incomplete subtypes (4). The underlying pathogenesis is unknown (1, 5). The reported cases are thought to be sporadic and might be due to the differentiation or migration of mesodermal structures during the early embryonic phase (4, 6). The first clinical feature of pentalogy of Cantrell is a cardiac malformation, and echocardiography has become the key to discovering this disease (1, 7, 8). Still, echocardiographers have an insufficient understanding of pentalogy of Cantrell, which may lead to missed diagnoses, especially in cases lacking the most obvious signs (9). Therefore, the present study presents a case of pentalogy of Cantrell, with a focus on the clinical and imaging findings.

## Case description

**Figure 1:**
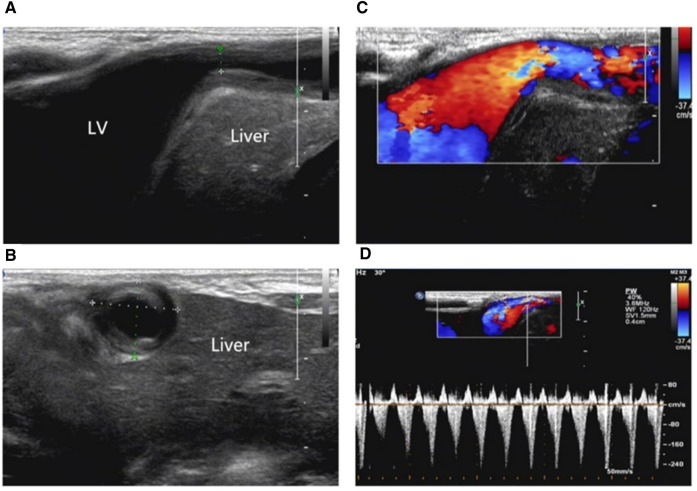
(**A**) Ventricular diverticulum in the front of the liver connecting to the apex of the left ventricle with a narrowed neck. (**B**) The left ventricular diverticulum is located between the liver and the anterior abdominal wall. (**C, D**). Blood flow signal in the diverticulum neck region.

**Figure 2:**
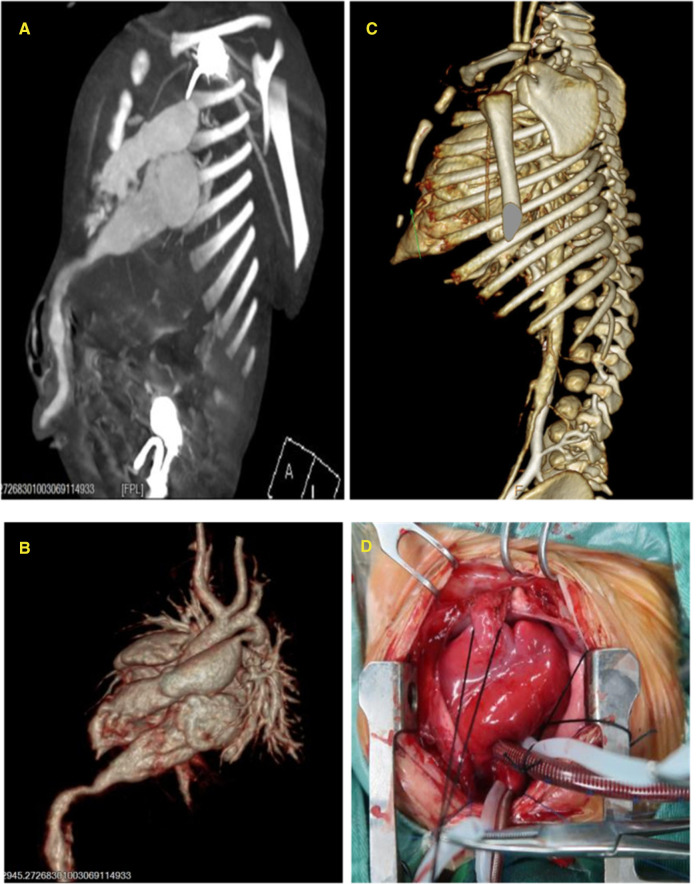
(**A**) Length of the ventricular diverticulum and narrowed neck connected to the left ventricle. (**B**) Three-dimensional reconstruction of the left ventricular diverticulum. (**C**) Partial defect of the lower sternum on computer tomographic angiography (CTA) (arrow). (**D**) Left ventricular diverticulum seen during operation.

One of twin male infants, at a gestational age of 37 weeks, was found with a cardiac murmur and a pulsatile mass in the midline supraumbilical abdomen for 2 days. The patient was admitted to the Affiliated Children's Hospital of Zhengzhou University in November 2016. The mother was para 1, gravida 1. The birth weight was 2.65 kg. There was no family history of genetic disease. A postnatal echocardiogram on day 2 showed cardiomegaly. Due to the possibility of congenital heart disease, the infant was transferred to our hospital. On admission, the heart was enlarged with an uplift of the anterior region. The cardiac sound was strong with a regular rhythm. The heart rate was 125 beats/min. A cardiac murmur was detected in the 3–4 intercostal space and extensively spread. In addition, a pulsatile mass was detected in the midline supraumbilical abdomen. The electrocardiography (ECG) axis was skewed to the left, and the T wave was low and inverted on the I and avL guides. Two atrial septal defects (ASD) measuring 6.8 and 2.0 mm, respectively, from the left to right shunt, a large ventricular septal defect (VSD) measuring 5.7 mm from the left to right shunt, a persistent ductus arteriosus (PDA), and severe tricuspid regurgitation were observed. A left ventricular diverticulum (LVD) herniating into the abdomen through the deficiency of the diaphragm and showing an affluent flow signal was located between the liver and anterior abdominal wall, as detected on color Doppler ultrasound. The diagnoses of LVD, ASD, VSD, PDA, severe tricuspid regurgitation, and pulmonary arterial hypertension were based on the echocardiogram findings (Figure 1A–D). The sternal defect and ventricular diverticulum were revealed using computer tomographic angiography (CTA) (Figure 2A–C). The male infant was diagnosed with pentalogy of Cantrell based on the ultrasound and CTA.

The patient was administered general anesthesia at a low temperature, and heart deformity surgery was performed under extracorporeal circulation. During the operation, the heart was rotated anticlockwise and enlarged. The left ventricle was found in the anterior and left sides. The pericardium defect was found in the apex of the left ventricle. The tubular structure (diverticulum), measuring 10 mm in the midline, extruded from the left ventricle via the pericardium defect. Then, it descended into the abdominal cavity behind the sternum through the diaphragm defect and towards the umbilical region. Partial muscle deficiency was found in the upper anterior abdominal wall. The diverticulum was cut off at the diaphragm, the proximal part of the diverticulum was sutured continuously, and the distal part was ligated. Subsequently, patch closures of ASD, VSD, and PDA ligation were performed. The pericardium and anterior diaphragm defects were also sutured (Figure 2D). The ECG at 6 days after surgery showed that the four cardiac chambers had shrunk, and the inner diameter of the pulmonary artery was decreased and had a normal pressure. The echocardiogram review in March 2018 showed that the heart structure and heart function of the child were normal.

## Discussion

The reported case showed that intracardiac anomaly and ventricular diverticulum are the primary manifestations of pentalogy of Cantrell. Pentalogy of Cantrell may be diagnosed by combining the ultrasound and CTA findings. The results may provide a reference for the recognition and diagnosis of Cantrell's pentad.

Cardiac anomaly, intracardiac anomaly, and ventricular diverticulum are the most common and primary manifestations of pentalogy of Cantrell and are correlated with the prognosis (1, 4). The intracardiac anomaly can be manifested as VSD, ASD, double outlet right ventricle, and other complicated intracardiac abnormalities. The ventricular diverticulum connects to the apex of the left ventricle or both the left and right ventricles (1). Due to the lower resistance of deficiency of the diaphragm, the heart grows towards the defect region and rotates clockwise, which could explain the formation of the diverticulum (10). The diverticulum extending through the diaphragm into the abdomen is evidence of organ deficiency; thus, a pulsatile mass could be detected in the upper abdomen. Intracardiac anomalies and diverticulum are detected on the echocardiogram. The supraumbilical abdominal defect shows a thin abdominal wall and a lack of partial anterior abdominal muscle and is usually accompanied by an omphalocele. The anomaly of the lower sternum is manifested as a sternal defect or a “V”-shaped cleft or complete cleft (11). Although CT can assist in evaluating the thoracic contour, the diagnosis of the defect of pericardium based on the imaging findings is difficult but can be detected intraoperatively. CTA also plays an important role in evaluating the abnormal heart and blood vessel structures. Therefore, combining ultrasound and CTA could improve preoperative planning (12).

In conclusion, intracardiac anomaly and ventricular diverticulum are the primary manifestations of pentalogy of Cantrell. The findings of the color Doppler echocardiogram are characteristics for the diagnosis of pentalogy of Cantrell. Therefore, it may be the first-choice modality for the evaluation of pentalogy of Cantrell due to its convenience, easy operation, and high resolution.

## Data Availability

The original contributions presented in the study are included in the article/Supplementary Material, further inquiries can be directed to the corresponding author/s.
